# Toxicology and pharmacology of botulinum and tetanus neurotoxins: an update

**DOI:** 10.1007/s00204-022-03271-9

**Published:** 2022-03-25

**Authors:** Marco Pirazzini, Cesare Montecucco, Ornella Rossetto

**Affiliations:** 1 Department of Biomedical Sciences, University of Padova, Via Ugo Bassi 58/B, 35131 Padova, Italy; 2 Centro Interdipartimentale di Ricerca di Miologia, CIR-Myo, University of Padova, Via U. Bassi 58/B, 35131 Padova, Italy; 3 Institute of Neuroscience, National Research Council, Via Ugo Bassi 58/B, 35131 Padova, Italy

**Keywords:** Tetanus, Botulism, Neurotoxins, Toxicity, Cholinergic, Therapy

## Abstract

Tetanus and botulinum neurotoxins cause the neuroparalytic syndromes of tetanus and botulism, respectively, by delivering inside different types of neurons, metalloproteases specifically cleaving the SNARE proteins that are essential for the release of neurotransmitters. Research on their mechanism of action is intensively carried out in order to devise improved therapies based on antibodies and chemical drugs. Recently, major results have been obtained with human monoclonal antibodies and with single chain antibodies that have allowed one to neutralize the metalloprotease activity of botulinum neurotoxin type A1 inside neurons. In addition, a method has been devised to induce a rapid molecular evolution of the metalloprotease domain of botulinum neurotoxin followed by selection driven to re-target the metalloprotease activity versus novel targets with respect to the SNARE proteins. At the same time, an intense and wide spectrum clinical research on novel therapeutics based on botulinum neurotoxins is carried out, which are also reviewed here.

## Introduction

Tetanus and botulism are human and animal diseases caused by protein neurotoxins released by sporogenic bacteria of the genus *Clostridium*. Clostridia are present in anaerobic parts of the environment and of the animal intestine, mainly in the form of spores. Under appropriate conditions, the spores germinate and the bacteria may produce the neurotoxins (Johnson and Montecucco 2008). Few isoforms of tetanus neurotoxin (TeNT) and many isoforms of botulinum neurotoxins (BoNTs) are known and are grouped in one TeNT serotype and in 8 BoNT serotypes (indicated by letters A, B, C, D, E, F, G and X). Serotypes include many isotypes differing in amino acid sequence impacting on their neurotoxicity and neutralization of polyclonal and monoclonal antibodies of serotyping antisera (isotypes are indicated by arabic numbers that follow the serotype capital letter) (Smith et al. 2005; Peck et al. 2017; Rossetto et al 2014; Maslanka et al. 2015; Peck et al. 2017; Azarnia Tehran and Pirazzini 2018; Dong et al 2019).

The few TeNT isoforms and the many BoNT isoforms presently known, are very similar in terms of amino acid sequence, 3D structure and biochemical mechanism of action within neurons. Yet, TeNT and BoNTs cause two different forms of neuroparalysis, spastic and flaccid respectively, because they act on different types of neurons: TeNT paralyzes central inhibitory interneurons of the spinal cord whilst the BoNTs paralyze peripheral cholinergic neurons and may affect central cholinergic neurons.

Tetanus is so evident that it was described at the beginning of medical literature by *Hyppocrates* who first defined the cardinal symptoms of the spastic paralysis of tetanus (Pappas et al 2008). Botulism symptoms are less evident and begin with cranial nerve palsy, with eyelid drop with blurred vision and diplopia followed by impaired swallowing. Paralysis then progressively descends to skeletal and respiratory muscles, including the diaphragm, leading to death (Sobel 2005). Respiratory and skeletal muscle paralysis is accompanied by impairment of autonomic cholinergic nerves and of the myenteric nervous system with associated symptoms. Mild forms of botulism frequently go undetected and even more severe forms of botulism may be diagnosed too late (Sobel 2005; Fleck-Derderian et al. 2017; Marlow et al. 2021; Rao et al. 2021). Although neuroparalysis may be so extensive to become lethal, BoNTs, as well as TeNT, do not kill the intoxicated neurons and, if the patient overcomes the respiratory deficit by mechanical ventilation, he/she will eventually recover, more or less completely. In fact, neurotoxins inside neurons have a limited lifetime that depends on several factors, including neuronal sensitivity, animal species, and neurotoxin susceptibility to intracellular protein degradation systems. In general, the time course of paralysis in humans spans from the 2 weeks of BoNT/E1 to the 3–4 months of BoNT/A1 (Megighian et al. 2021; Rao et al. 2021; Rossetto et al. 2014).

Tetanus and botulism are rare diseases owing to a very immunogenic tetanus vaccine (tetanus toxoid, i.e., formaldehyde detoxified TeNT, plus Aluminium oxide adjuvant) and to improved preparation and preservation of foods that prevent alimentary botulism. This is the most common form of botulism, followed by infant botulism which is characterized, together with intestinal adult botulism, by the clostridial colonization of the intestine (Fenicia and Anniballi 2009; Rao et al. 2021).

## Structure and mechanism of action of TeNT and BoNTs

**Fig. 1:**
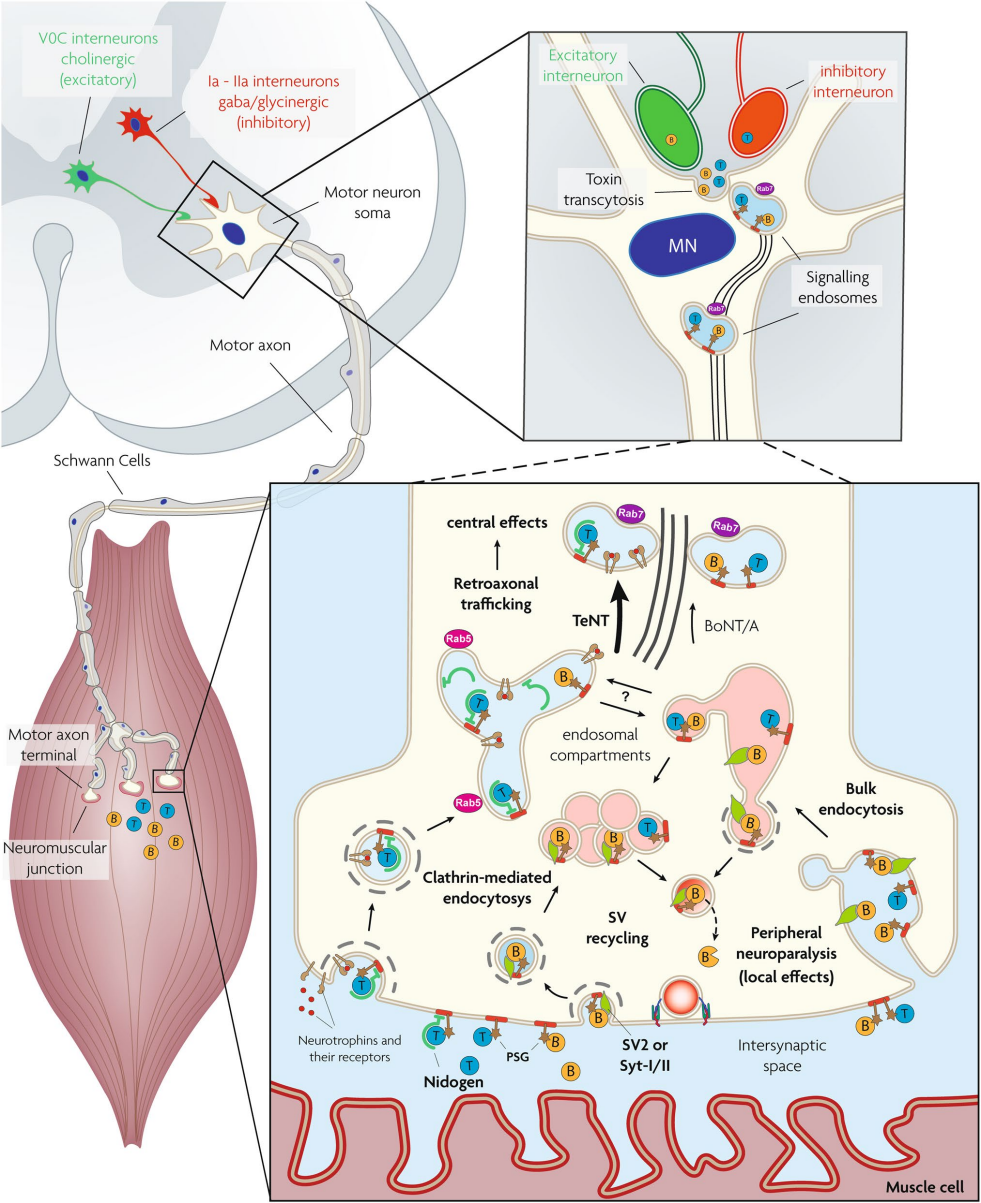
Internalization and trafficking of TeNT and BoNTsinside peripheral neurons. *Bottom left panel*: TeNT (light blue circle) and BoNTs (orange circle) bind to peripheral nerve terminals, most notably the motor axon terminals of the neuromuscular junction. Although TeNT binds also other types of nerve terminals, BoNTs bind selectively motorneurons and autonomic cholinergic nerves. *Bottom right panel*: TeNT binds via interaction with a polysialoganglioside (brown star) and nidogen molecule (green). BoNTs bind to a polysialoganglioside and to a protein receptor (SV2 or synaptotagmin, green). The TeNT-receptors complex enters the lumen of an endocytic vesicle that later on merges with a signalling endosome (SE) distinguished by the rab5 molecule (magenta). The replacement with rab7 (violet) determines the attachement of SE to the machinery (black tubules) that mediates their retroaxonal transport to the motorneuron perikaryon located within the spinal cord as shown in the *top right panel*. The BoNT-receptors complexes enter the lumen of a synaptic vesicle that leads to the translocation of the catalytic L chain in the cytosol of the presynaptic terminal (see Fig. 2 for more details), causing the local neuroparlaysis of botulism. Also the TeNT trimeric complex may enter the lumen of a synaptic vesicle and have a local effect, albeit to a much lesser extent than the BoNTs Similarly, BoNT/A is retraxonally transported as TeNT does, although the proportion and the specific trafficking route(s) are still unknown *Top left panel*: TeNT and BoNTs are discharged in the spinal cord fluids and bind inhibitory interneurons (Ia and IIa, glycinergic and gabaergic) or VoC cholinergic excitatory interneurons, respectively. As a consequence a spastic paralysis is caused by TeNT, whilst the action of BoNT contributes to the peripheral motorneurons’ paralysis

**Fig. 2:**
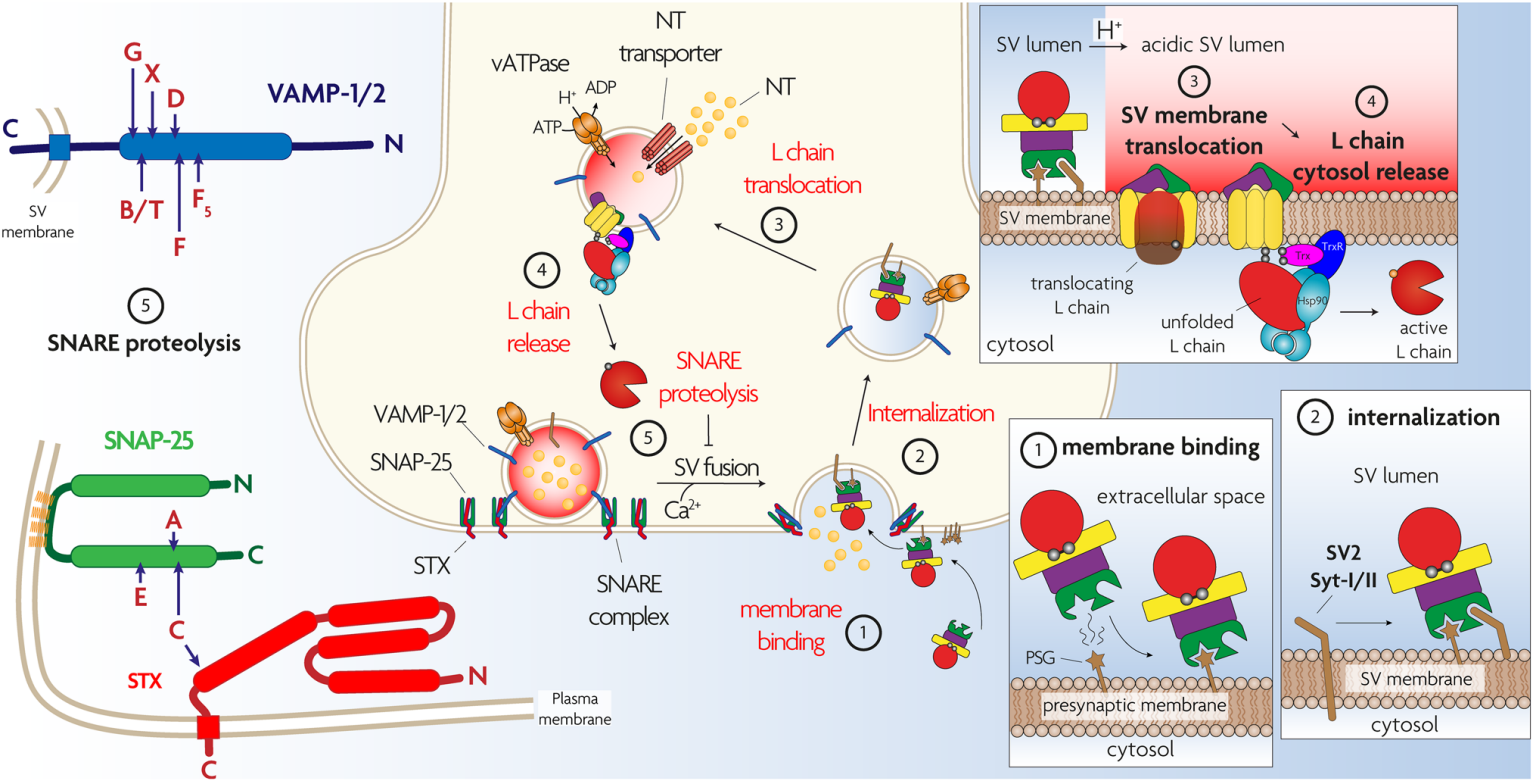
The five steps of the molecular mechanism underlying the neuroparalytic action of tetanus neurotoxin (TeNT) and botulinum neurotoxins (BoNTs). *Step 1*: the neurotoxins bind to a polysialoganglioside molecule (brown star) and then to a protein receptor (detailed cartoons in the bottom right of the figure) via the C-terminal domain of the toxin (green). *Step 2*: this second binding drives the trimeric complex into the lumen of a synaptic vesicle. The vesicle is acidified (transition from light blue to red in the upper right panel) following the action of a proton pump (orange) to drive the accumulation of neurotransmitter (NT, pale orange) inside the synaptic vesicle. *Step 3*: low pH causes a structural transition of th neurotoxin that results in the membrane penetration of the HN domain (yellow) and translocation of the metalloprotease domain (red), which remains bound to HN via a disulfide bond (upper right panel). Translocation is assisted by the chaperone Hsp90 (light blue). *Step 4*: The disulfide bond is reduced by the NADH-Thioredoxin -Thioredoxin Reductase system (magenta and blue, respectively) (upper right panel). *Step 5*: the free metalloprotease domain reaches its specific SNARE protein target which is cleaved and inactivated by a single site cleavage, in such a way that neurotransmitter release from the synaptic vesicle is prevented. The left panel shows that TeNT cleaves only VAMP, whilst the various BoNT serotypes target VAMP or SNAP25 or Syntaxin (STX) at specific peptide bonds

TeNT and BoNTs consist of a light chain (L, 50 kDa) and a heavy chain (H, 100 kDa) linked by a single disulfide bond and folded in four domains, each one playing a specific role in the intoxication of nerve terminals. The L chain folds in the N-terminal domain and it is a Zn^2+^-dependent endopeptidase specific for one or more of the three SNARE proteins (VAMP, SNAP-25, syntaxin). These proteins form a heterotrimeric SNARE complex which is the core of the nanomachine that mediates neuroexocytosis and neurotransmitter release (Pantano and Montecucco 2014; Pirazzini et al. 2017; Dong et al. 2019). The L domain is encircled by a peptide belt formed by domain HN (N-terminal part of the H chain, 50 kDa). This domain is characterized by two long α-helices and by additional shorter helices located around the interchain disulfide bond. HN assists the translocation of the L domain into the cytosol (Fischer and Montal 2007, 2013; Pirazzini et al. 2017). HN is linked to the carboxy terminal 50 kDa part of H which consists of two domains termed HC-N (25 kDa) and HC-C (25 kDa). HC-N is essential for neurotoxicity, although its exact role has not been clarified and might be different for TeNT and BoNT serotypes (Deppe et al. 2020). There is evidence that HC-N is involved in toxin binding by interacting with negatively charged lipid microdomains (Muraro et al. 2009; Zhang and Varnum 2012). At variance, HC-C is responsible for the exquisite neuroselectivity of TeNT and BoNTs and for their different intraneuronal trafficking that is responsible for the peripheral activity of BoNTs and the central one of TeNT which follows its peripheral uptake and retroaxonal transport to the spinal cord (Dong et al 2019; Surana et al. 2018; Sleigh et al 2020; Megighian et al. 2021). As shown in Fig. 1, these different trafficking pathways of TeNT and BoNT inside neurons are not mutually exclusive as TeNT can cause local flaccid paralysis and BoNTs can migrate retrogradely inside neurons and be released in the CNS at various levels (Grumelli et al 2005; Mazzocchio and Caleo 2015; Caleo et al. 2018; Megighian et al. 2021). Both TeNT and BoNTs within their specific presynaptic nerve terminal targets act with a similar mechanism, strictly linked to their modular structure, that consists of five main steps, as summarized in Fig. 2: (1) presynaptic membrane binding, (2) endocytosis inside synaptic vesicles, (3) membrane translocation of the L domain to the cytosol assisted by domain HN, (4) reduction of the interchain disulfide bond with activation of the L metalloprotease domain, (5) selective cleavage of one or more of the three SNARE proteins with consequent blockade of neurotransmitter release.

### Step 1. Neuro-selective binding to peripheral nerve terminals

TeNT and BoNTs bind with high selectivity and affinity to the presynaptic plasma membrane via a sequential interaction with polysialogangliosides (PSGs) highly enriched in nerve terminals and protein receptors (Rummel 2015; Surana et al. 2018). PSGs have a large oligosaccharide portion, including several negatively charged sialic acid residues, that projects out from unmyelinated surfaces of the presynaptic neuronal membrane making it ideal to bind large proteins such as the neurotoxins or immunoglobulins specific for oligosaccharides (Chiba et al. 1992; Rossetto et al. 2014). This process is facilitated by the fact that PSG are negatively charged and embedded within membrane patches containing anionic lipids (Simons and Toomre 2000; Prinetti et al. 2009), whilst TeNT and BoNT proteins are dipoles whose cathode is centered in their PSG binding pocket. This orients the BoNT molecule while approaching the plasma membrane thus favoring their landing with the HC-C domain and increasing the probability of membrane binding (Fogolari et al. 2009). The role of PSGs as primary receptors is supported by several experimental evidence (Binz and Rummel 2009), and by the fact that the most TeNT- or BoNT-neutralizing monoclonal antibodies sterically prevent PSG binding (Garcia-Rodriguez et al. 2007, 2021; Mukherjee et al. 2012; Lam et al. 2018; Tomic et al. 2021; Pirazzini et al. 2021; Wang et al. 2016, 2021; Zhang et al. 2021; Brier et al. 2021).

### Step 2. Internalization and trafficking

PSG-bound TeNT and BoNTs are free to diffuse within the two-dimensional lipid solvent of the presynaptic membrane strongly increasing the probability to meet their second receptor. Extensive evidence shows that BoNTs bind the luminal domain of an integral protein present on the membrane of synaptic vesicles (SV). This SV receptor protein is either synaptotagmin-1/2 for BoNT/B, BoNT/DC, BoNT/G or SV2 for BoNT/A, BoNT/E and TeNT (Dong et al. 2019). The luminal protein receptor domains become transiently available for toxin binding after SV fusion with the presynaptic membrane and neurotransmitter release (Binz and Rummel 2009; Rossetto et al. 2014; Rummel 2015; Pirazzini et al. 2017; Dong et al. 2019). Following this interaction, the neurotoxins are internalized inside the SV lumen (Harper et al. 2011, 2016; Colasante et al. 2013). Recently, the elegant use of knock-in mouse models expressing either synaptotagmin-1 or -2 with specific point-mutations that disrupt BoNT neuronal entry, has shown the primary role of synaptotagmin-1 in mediating the neuroparalytic effects of BoNT/B, /G and /DC in autonomic nerves and of synaptotagmin-2 in the neuromuscular system (Thaker et al. 2021). These results contribute to account for the fact that BoNT/B requires higher doses to be effective in the therapy of hyperactivities of human NMJ with respect to BoNT/A1 (Guidubaldi et al. 2011; Bentivoglio et al. 2015). Accordingly, BoNT/B mutants designed to improve the recognition of human synaptotagmin-2 were found to strongly enhance the therapeutic potential of BoNT/B (Tao et al. 2017).

BoNT/A and /E bind the glycosylated fourth luminal loop of SV2 via interaction with the polypeptide chain and the glycan portion (Benoit et al. 2014; Yao et al. 2016). The glycan binding may explain the fact that BoNT/A1 has slightly different potency in different patients as protein glycosylation is known to vary substantially among human individuals (Montecucco and Zanotti 2016).

### Retroaxonal transport to the Central Nervous System

The protein receptor responsible for the retroaxonal transport of TeNT remained elusive for long time. Initial findings documented TeNT entry inside a pool of endocytic vesicles referred to as signalling endosomes, which integrate clathrin-mediated endocytosis with the sequential activity of Rab5 and Rab7 GTPases to send neurotrophic signals from the periphery to the soma of peripheral neurons (Deinhardt et al. 2006). More recently, experimental evidence indicated that nidogen-1 and nidogen-2, two proteins that interact reversibly with the basal lamina, act protein receptors that, together with PSG, are responsible for directing TeNT into signaling endosomes (Bercsenyi et al. 2014; Debaisieux et al. 2016; Gibbs et al. 2016; Sleigh et al 2019, 2020). These organelles move retroaxonally up to the perikaryon and release TeNT into the spinal cord fluid close to the presynaptic membrane of inhibitory interneurons which then bind and endocytose the toxin inside SV, similarly to BoNTs (Matteoli et al. 1996). One report indicated that SV2 might mediate this process (Yeh et al. 2010), though this remains controversial (Blum et al. 2012).

Initial evidence that also BoNT/A1 is retroaxonally transported from peripheral nerve terminals to the ventral roots of the spinal cord were based on isotopically labelled neurotoxin (Habermann 1974; Wiegand et al. 1976) and on indirect neurophysiological observation (Currà et al. 2004; Berardelli and Conte 2021). Additional recent experimental evidence, based on imaging methods, indicated that BoNT/A1 migrates retrogradely inside axons and moves by transcytosis events to various parts of the CNS where it paralyses nerves by cleaving SNAP-25 (Antonucci et al. 2008; Caleo and Schiavo 2009; Matak et al. 2011; Restani et al. 2012; Mazzocchio and Caleo 2015; Caleo et al. 2018; Weise et al. 2019). Notably, truncated SNAP-25 appeared in: (i) the facial nucleus and in the trigeminal nucleus caudalis of rats after injection of BoNT/A1 in the whisker pad (Antonucci et al. 2008; Matak et al. 2011, 2014), (ii) the ventral horns of the spinal cord upon injection in the rat hind-limb (Matak et al. 2012) and (iii) the dorsal horn after peripheral subcutaneous injection in the hind paw or intramuscular injection in the hindlimb (Marinelli et al. 2012). Noteworthy, SNAP-25 cleavage was found in neurons that are even two synapses away from the neuron entered at the injection site (Caleo et al 2018) indicating the ability of the toxin to undergo sequential cycles of neuronal entry, axonal transport and transcytosis.

The specific interactor(s) and the internalization route responsible for BoNT/A retroaxonal movement remains poorly understood. There is evidence that BoNT/A1 is co-transported with TeNT in cultured motor neurons in vitro, suggesting that BoNT/A enters non-acidic carriers as TeNT does (Restani et al. 2012). Possibly, BoNT/A1 enters different SV pools at the NMJ (Harper et al 2011, 2016). It has been suggested that BoNT/A1 also enters autophagosomes generated at synaptic contacts in an activity dependent manner and is then retrotrasported to the soma (Wang et al. 2015). However, this latter pathway includes lumenal acidification and therefore is unlikely to be relevant for the eventual entry into second order neurons (see next paragraph). There is evidence that BoNT/A1 that has reached spinal cord fluids enters and cleaves SNAP-25 into cholinergic synapses directly impinging on motor neurons, possibly corresponding to the c-boutons of V0_c_ interneurons (Caleo et al. 2018). These interneurons mediate excitatory inputs to facilitate the excitability of α-motor neurons and to modulate the extent of their activation, i.e., their firing at the NMJ (Caleo and Restani 2018; Mazzocchio and Caleo 2015; Caleo et al. 2018). The activity of BoNT/A in these interneurons could in part explain the persistent therapeutic efficacy of BoNT/A1 for different neurologic conditions including dystonia and spasticity after cerebral stroke (Mazzocchio and Caleo 2015).

### Step 3. Membrane translocation

After neurotransmitter release, SV are retrieved from the plasma membrane and refilled with neurotransmitters, and this latter process is driven by the transmembrane pH gradient generated by the vesicular ATPase proton pump that acidifies the SV lumen. Lumenal low pH is exploited by TeNT and BoNTs to translocate their L chain into the cytosol, via a low-pH-induced conformational change of the toxin molecule. This involves the HN domain, which inserts into the SV membrane, forms an ion channel and assists the translocation of the L domain to the cytosolic side of the SV membrane (Fischer and Montal 2013; Pirazzini et al. 2017). Exactly how this happens is not yet known. Two models have been proposed that differ for a different role played by the HN transmembrane channel (Pirazzini et al. 2017; Dong et al. 2019). One suggests that the formation of a HN channel is a pre-requisite for L translocation, i.e., the L chain passes through a pre-formed HN channel (Montal 2014; Fischer and Montal 2007, 2013). The other model suggests that the channel is formed during (or after) the passage of the L chain across the membrane, i.e., the HN channel is a consequence of L translocation (Pirazzini et al. 2015b, 2017).

Computational and mutagenesis studies have led to the identification of three carboxylate residues that are very likely to be involved in the low pH driven structural transition of BoNT/B (Pirazzini et al. 2013a). More recently, a structural motif containing several carboxylate residues, present in HN, potentially involved in the same process was identified in BoNT/A, close to the SS bond (Lam et al. 2018). This motif includes some disordered loops and short α-helices that rearrange at acidic pH values into hydrophobic β strands with membrane insertion ability. This “BoNT-switch” has been suggested to be one of the first parts of HN inserting into the membrane, a process which might work in concert with the membrane penetration of the hydrophobic SS interchain bond (Pirazzini et al. 2017). Interestingly, a similar low pH switch motif was recently identified in TeNT (Pirazzini et al. 2021), suggesting that this structural–functional motif is conserved among different clostridial neurotoxins and plays an important role in L translocation. This is further indicated by the fact that monoclonal antibodies against the BoNT/A- and TeNT-switch block the L chain translocation and display a very potent antitoxin activity (Dolimbek et al. 2007; Pirazzini et al. 2021). Another important part of TeNT involved in the L chain membrane translocation is the charged loop ^767^DKE^769^ connecting α15 and α16 (*cis*-loop) as its aliphatic substitution inhibited LC translocation without affecting binding, intracellular trafficking and or HN-mediated pore formation. K^768^ is conserved among the CNTs and these results implicate the *cis*-loop in domain L translocation, independently of pore formation (Zuverink et al 2020).

### Step 4. Reduction and cytosolic release of the metalloprotease L domain

During and after translocation, the L domain has to unfold/refold and Hsp90, a major cytosolic chaperone involved in the folding of nascent proteins, was found to be implicated in this process; accordingly, specific Hsp90 inhibitors prevent the intoxication of nerve terminals caused by TeNT and BoNTs (Pirazzini et al. 2018; Azarnia Tehran et al. 2017). On the cytosolic surface of SV, the metalloprotease L domain remains attached to the H chain via the interchain SS bond, which has to be reduced to enable the enzymatic activity (Schiavo et al. 1992b). Out of the several redox couples acting in the cell cytosol, the NADH-Thioredoxin Reductase-Thioredoxin intracellular redox system (TrxR-Trx) was found to be present on the cytosolic side of the SV membrane (Pirazzini et al. 2014, 2015a). TrxR-Trx interacts with Hsp90 and releases the L activity (Azarnia Tehran et al. 2017). Specific inhibitors of this redox system prevent tetanus and botulism in mice and are pan-inhibitors acting on all BoNT serotypes (Pirazzini et al. 2013b, 2014; Zanetti et al 2015, 2021; Pirazzini and Rossetto 2017; Rossetto et al 2019a, b).

### Step 5. Metalloproteolytic cleavage of SNARE proteins with blockade of neurotransmitter release

Once released into the neuronal cytosol, the L chain cleaves one or more of the three SNARE proteins: VAMP is an integral protein of the SV membrane, whilst SNAP-25 and syntaxin are on the cytosolic surface of the presynaptic membrane. They form a heterotrimeric complex which is the core of the membrane fusion nanomachine allowing the release of the neurotransmitter in the intersynaptic space (Jahn and Scheller 2006). The discovery that TeNT and BoNTs cleave SNAREs preventing neuroexocytosis provided the strongest experimental evidence for the essential role of SNAREs in neuroexocytosis (Pantano and Montecucco 2014). The SNARE specificity of the L chain metalloproteases is based on their recognition of multiple sites of their substrates (Rossetto et al. 1994; Brunger and Rummel 2009; Binz 2013; Gardner and Barbieri 2018), which makes more difficult to identify specific drug inhibitors (see below).

Of note, the specific cleavage of SNARE proteins has become a “refence feature” to classify novel BoNTs and BoNT-like toxins, particularly those identified by genome mining (Doxey et al. 2018). The first one was identified by computational methods in the genome of *Weissella oryzae*, a lactobacillus growing in fermented rice (Mansfield et al. 2015). Its gene sequence has strong similarities with bont genes and its L metalloprotease was found to fold similarly to BoNT/B, and to cleave VAMP close to its transmembrane domain (Zornetta et al. 2016). A novel ***bont*** gene was similarly identified in the genome sequence of a *C. botulinum* strain associated to a case of infant botulism and the recombinant protein toxin was not recognized by any antiserum and proposed to be a novel serotype dubbed BoNT/X (Zhang et al. 2017). Its protease activity **is** unique in terms of peptide bond and VAMP isoform cleaved including VAMP isoforms not involved in neuroexocytosis. More recently, the analysis of the genome of an *Enterococcus* species revealed a *bont* gene similar to BoNT/X termed BoNT/En, and its metalloprotease cleaved all three SNARE proteins suggesting a novel form of target recognition (Brunt et al. 2018; Zhang et al. 2018). A similar computational analysis identified of *Chryseobacterium piperi* a *bont* gene encoding for a BoNT-like protein endowed with all the active site residues essential for the metalloprotease activity, but of unknown substrate (Mansfield et al. 2019). In 2019, the first BoNT that specifically paralyses insects was found to be produced by *Paraclostridium bifermentans* and dubbed PMP1. The L domain of PMP1 cleaves anopheline mosquito syntaxin, but not mammalian syntaxins (Contreras et al. 2019). Such a finding was predicted on the basis of considerations on BoNTs and their ecological chains (Montecucco and Rasotto 2015), but the ecological role of the anopheles specificity of PMP1 is still to be found. It is safe to speculate that many novel insect-specific BoNT-like toxins will be found by computational studies of the pangenome similalirly to what found for viruses (Edgar et al. 2022).

## Toxicology of tetanus and botulinum neurotoxins

These neurotoxins affect different parts of the nervous system whose complete functionality is required for survival. In all cases, they deliver inside their target neurons a metalloprotease that specifically cleaves one copy after the other of proteins essential for neurotransmission. The combination of neurospecificity, enzymatic activity and importance of the target neurons for survival makes them the most powerful poisons for mammals. Their toxicity varies with the isoform of BoNT considered and with the entry route. The oral and respiratory routes are less efficient than the intramuscular, intravenous or i.p. routes. Only one serotype of TeNT is known and the lowest i.p. mouse LD50 value is around 0.2 ng/Kg which corresponds to a femtomolar concentration assuming an even distribution in circulating fluids; the similar manifestation of symptoms of tetanus in monkeys and human patients indicate that a similar toxicity may be extrapolated from monkey to humans (Rossetto and Montecucco 2019).

A comprehensive analysis of the available literature has recently reported mouse LD50 values comprised between 0.02 and 5 ng/Kg for BoNTs (Rossetto and Montecucco 2019). The 0.02 ng/Kg (i.p.) of recombinant BoNT/D in mice is at large variance with the low toxicity of BoNT/D in humans (Eleopra et al. 2013) illustrating the animal species dependency of the toxicity of TeNT and BoNTs. This problem has been studied extensively in the past for TeNT (Wright 1955; Rossetto and Montecucco 2019), but a comparable set of data is still lacking for BoNTs. In fact, only few of the many dozens of BoNT isoforms have been studied in terms of toxicities in different animals, also because many of them have been identified only by computational genomics (Doxey et al. 2018). The evolution and selection of many BoNTs are likely to be strictly related to the specific physiology and ecology of each animal species, including invertebrate animals (Montecucco and Rasotto 2015).

### Inhibitors of the toxic action of TeNT and BoNT

The extreme toxicity of BoNTs for humans has led to the inclusion of botulinum neurotoxins in the first category of the tier 1 list of potential agents for bioterrorism (Arnon et al. 2001; Villaer et al. 2006; Bhattacharjee 2011; Cenciarelli et al. 2019). TeNT has not been considered for such a use because of the worldwide prophylaxis with the potent anti-tetanus vaccine and antisera. Accordingly, an intense research for active site inhibitors started right at the time of the discovery of the metalloprotease activity of TeNT and BoNT (Schiavo et al. 1992a, b). In the search of inhibitors of BoNTs, one can distinguish four levels of investigation of increasing complexity: (A) in vitro inhibitors of the metalloproteolytic cleavage of the SNARE protein substrate; (B) metalloprotease inhibitors inside neurons in culture; (C) drugs preventing botulism in animals; (D) drugs that prevent the action of BoNT in humans. Peripheral neuroparalysis in animals and humans is the final result of a series of events that begin with absorption of BoNTs at the intestinal or respiratory levels or with injection, followed by diffusion in the body via the lymphatic and blood circulations that deliver the neurotoxins to nerve terminals. Inhibition of neurotransmitter release occurs following the five steps described above, namely: (1) neuronal binding, (2) internalization, (3) membrane translocation, (4) release of the metalloprotease domain, (5) SNARE cleavage with block of neurotransmitter release.

The first group of inhibitors of tetanus and botulism were polyclonal, and later, monoclonal antibodies, which act by interacting with the neurotoxins and neutralizing their activity, in most cases by preventing their binding to neurons. In general, antibodies do not neutralize the neurotoxins once they are hidden inside neurons. Therefore, anti-neurotoxin antibodies are effective when injected before symptoms of tetanus and botulism appear. Afterwards, toxin-neutralizing antibodies are still useful because they neutralize circulating neurotoxin that is detectable long after disease onset, and that may extend and expand symptoms aggravating the disease.

A particular case is that of tetanus whose symptoms are the result of the TeNT action on inhibitory interneurons of the spinal cord. TeNT is transported from the periphery to the CNS inside endosomes which move within neuronal axons, and it is, therefore, well protected from antibody binding (Schmieg et al. 2014). Accordingly, therapy by intratechal administration of antibodies is more efficacious than intravenous infusion because the TeNT binding antibodies are infused in the anatomical site of action of this neurotoxin (Kabura et al. 2006). Research on anti-TeNT and anti-BoNTs antibodies has been discussed in previous reviews and papers (Chen et al 2012; Mukherjee et al. 2012; Rasetti- Escargueil and Popoff 2019; Garcia-Rodriguez et al. 2007, 2021; Lam et al. 2020; Snow et al. 2019; Brier et al. 2021; Tomic et al. 2021), and we refer to them and to references cited therein, whilst only more recent reports are discussed here.

In the case of tetanus, recent research has focused on human monoclonal antibodies (humAbs) anti-TeNT (Wang et al. 2016, 2021; Aliprandini et al. 2019; Ghotloo et al. 2020; Minamitani et al. 2021; Pirazzini et al. 2021; Zhang et al. 2021) with the aim of overcoming some clinical problems encountered with the use of the IgG fraction isolated from human polyclonal antisera derived from tetanus toxoid immunized donors (Pirazzini et al. 2021). The toxin neutralizing humAbs were identified using phage display libraries or by screening antibody producing cells isolated from human individuals that were treated in adult age with the tetanus toxoid vaccine. The isolated humAbs recognize different epitopes in different domains of TeNT with variable affinities. Most toxin neutralizing humAbs bind the HC receptor binding part of TeNT thus preventing binding (step 1), but one of them recognized domain HN preventing step 3) (Pirazzini et al. 2021). One extremely powerful neutralizing humAb was found to bind domain HC-C of TeNT via an epitope that contains both toxin receptors, resulting in a picomolar dissociation constant and in the neutralization of TeNT by stoichiometric amounts of its Fab derivative (Pirazzini et al. 2021).This Fab was suggested to have a strong therapeutic potential via the intratechal route as it overcomes the limitation of the small amount of proteins that can be safely administered via this route. The humAbs anti-TeNT tested so far have addressed levels of investigation A, B and C but none has yet been tested in humans (level D).

Extensive work has also been conducted to elaborate a serotherapy for BoNTs, an approach which is complicated by the existence of many isoforms and of several serotypes of BoNTs, with respect to the single serotype of TeNT. HumAbs as well as camelid single domain antibodies specific for different BoNT serotypes have been prepared and shown to prevent botulism when injected before or together with BoNTs. In addition, it appears that BoNTs are less immunogenic that TeNT and that combination of three/four antibodies are to be used to neutralize completely one BoNT serotype (Chen et al. 2012; Mukherjee et al. 2012; Garcia-Rodriguez et al. 2007, 2021; Lam et al. 2018; Snow et al. 2019; Tomic et al. 2021; and refences contained therein).

Antibodies cannot penetrate inside cells and therefore their neutralizing power is lost upon neurotoxin entry into neurons. However, two different reports have recently described an ingenious method to neutralize BoNT/A1 action inside neurons in vivo. This method is based on the generation of a chimeric construct consisting of a single chain lama antibody (sclAb) that specifically neutralizes the free form of the metalloprotease L domain of BoNT/A1, fused to N-terminus of an inactive mutant of BoNT/A1 (Miyashita et al. 2021) or BoNT/C1 (McNutt et al. 2021). These chimeras bind cholinergic neurons and deliver the sclAb in the nerve terminal cytosol, where the sclAb inactivates the metalloprotease domain of BoNT/A1 with restore of nerve to muscle signalling. These chimeric anti-BoNT/A1 constructs were tested at levels (A), (B) and (C) which included, in one case, guinea pigs and nonhuman primates (McNutt et al. 2021), in addition to mice (Miyashita et al. 2021).

One limitations intrinsic to such an approach, partially shared with BoNT serotherapy, is the existence of several BoNT serotypes, which requires the preparation of large stocks of many chimeric sclAB-BoNT. In addition, in the case of a botulism bioterrorist attack, the logistic associated to the effective delivery of the therapeutic protein chimeras in very large numbers of patients is bound to be very complicated. Moreover, the costs of the production and storage of large amounts of chimeric protein molecules are expected to be very high. However, the studies of McNutt et al. (2021) and Miyashita et al. (2021) provide a proof of principle of a novel therapeutic intervention of general value; one that can be extended to a variety of human diseases caused by pathogenic protein mutants present in the cytosol of neurons.

These problems do not affect small drugs targeted to steps of the mechanism of action of BoNT that are identical in all serotypes. However, none of the many molecules tested so far was shown to be active beyond levels (A) and (B). The majority of small molecules, peptides or peptidomimetics with a potential of becoming anti-botulism drugs, tested so far, are directed to step 5), i.e., the metalloprotease activity which eventually causes the flaccid neuroparalysis of botulism (Rossetto et al. 2014). Clearly, post-exposure drugs are the ones needed after appearance of botulism symptoms following poisoning with contaminated food, or improper use of therapeutical/cosmetic BoNT/A1 or following a bioterrorism attack (Arnon et al. 2001; Dressler and Benecke 2003; Yiannakopoulou 2015; Witmanowski and Błochowiak 2020).

The first group of small molecules considered were those known to inhibit other metalloproteases (thermolysin, angiotensin converting enzymes, matrix metalloproteinases inhibitors and peptides encompassing the VAMP cleavage site) but they proved to be rather ineffective (Schiavo et al. 1992a, b). The problem is complicated by the fact that TeNT and BoNT metalloproteases recognize their SNARE substrate via interaction with active site as well as with exosites (Brunger and Rummel 2009; Agarwal et al. 2009). Comprehensive specific reviews cover such studies (Li et al. 2010; Kiris et al. 2014; Lin et al. 2019, 2020). In general, up to the present time, inhibitors of the metalloprotease activity of BoNTs were found to be effective only at levels (A) and/or (B) with *K*_*i*_ or IC_50_ values in the 10^–6^ – 10 ^−8^ Molar range. (Caglič et al. 2014; Kumar et al. 2016; Bremer et al. 2017; Vieni et al. 2018; Patel et al. 2018; Garland et al. 2019; Amezcua et al. 2021; Lin et al. 2021; Turner et al. 2021a, b). None the less, these studies are very important because they provide the molecular basis to design novel and specific inhibitors of the BoNT active site acting in cultured neurons in the nanomolar range, a value which is a prerequisite figure to proceed to animal studies (level C). This result has not yet been achieved but there is no reason for it cannot be attained. A great impulse may derive from novel artificial intelligence approaches to drug-protein design to be elaborated on the basis of the breakthroughs in protein structure studies recently reported (Jumper et al. 2021; Tunyasuvunakool et al. 2021; Baek et al. 2021; Humphreys et al. 2021).

Meanwhile, other approaches to block TeNT and BoNTs neuron intoxication have been attempted by focusing on other steps of nerve terminal paralysis. The Trx-TrxR redox system-dependent release of the L chain from the H chain via disulfide reduction enables the display of the metalloprotease activity (Rossetto et al. 2019a, b). Consequently, a range of Trx-TrxR-specific inhibitors were tested as anti-botulism drugs, and Ebselen and PX-12 were found to fully prevent SNAREs cleavage caused by all serotypes of BoNT in cultured neurons at nanomolar concentration (Pirazzini et al. 2014; Montal 2014; Zanetti et al. 2015). Ebselen has been tested in human clinical trials for acute ischemic stroke and neurologic diseases (Yamaguchi et al. 1998; Ogawa et al 1999; Singh et al. 2013, 2016; Masaki et al. 2016a, b; Sharpley et al. 2020). In mice, Ebselen prevents the flaccid paralysis of botulism caused by any of the seven serotypes (A to G) tested at the dose of 7.5 mg/Kg (Zanetti et al. 2015). It has all the characteristics to be a potential candidate to prevent botulism and to treat infant and adult intestinal botulism which are characterized by the intestinal infection by *Clostridium botulinum* (or *barati* or *butirycum*) with continuous production of BoNT (Fenicia and Anniballi 2009; Rossetto et al 2014). One potential advantage of Ebselen derives from its ability to modify Cys165 which points its sulfur atom towards the zinc atom of the active site of BoNT/A1 and /F (Garland et al. 2019).

Another anti botulism drug not acting directly on the neurotoxin molecule, but on the probability of synaptic vesicle fusion with the presynaptic membrane is 3,4-Diaminopyridine (3,4-DAP). SNARE cleavage prevents SV fusion triggered by Ca^2+^ ions that are admitted in the cytosol via the voltage gated Ca^2+^ channels. 3,4-Diaminopyridine (3,4-DAP) increases Ca^2+^ cytosolic concentration within the presynaptic nerve terminal by blocking membrane potential controlling presynaptic K^+^ channels thus prolonging the action potential. Long ago 3,4-DAP was shown to relieve only BoNT/A induced paralysis, whilst being inactive on all other BoNT serotypes (Siegel et al. 1986; Simpson 1986; Molgo et al. 1987; Adler et al. 1995). This is most likely due to the unique cleavage sites of SNAP-25 by BoNT/A (Schiavo et al. 1993) as only the SNARE complex comprising BoNT/A-cleaved SNAP-25, lacking only nine residues from the C-terminus, appears to retain a partial Ca^2+^ sensitivity (Lawrence et al. 2002; Montecucco et al. 2005; Megighian et al. 2010). Recently, a re-purposal of 3,4-DAP for the treatment of botulism was advanced, based on the approval of 3,4-DAP by EMA and FDA for the treatment of the Lambert Eaton myasthenic syndrome (https://www.ema.europa.eu/en/medicines/human/orphan-designations/eu302124; https://www.accessdata.fda.gov/drugsatfda_docs/nda/2018/208078Orig1s000OtherR.pdf) and on a careful study of the activity of 3,4-DAP performed in a mouse model of botulism (Vazquez-Cintron et al. 2020).

Another anti-botulism druggable intracellular function is the degradation of the L domain by the ubiquitin proteasome pathway which determines the long duration of the paralysis caused by BoNT/A1 (Tsai et al. 2010, 2017). A very recent screening of a library of inhibitors focused on this pathway, performed on cultured motor neurons derived from mouse embryonic stem cell, led to the identification of several compounds capable of increasing the rate of degradation of the L domain of BoNT/A1; in addition, two deubiquitinases specifically involved in this protein removal were identified (Sen et al. 2021). It will be interesting to test these compounds in mice and on other BoNT serotypes.

## Pharmacology of botulinum neurotoxins

Soon after their discovery, BoNTs were considered as a potential military bioweapon because of the exceedingly high potency and they have been included among the Category A agents which possess high risk to national security by CDC (CDC National Select Agency Registry. http://www.selectagents.gov/). They are easily isolated as crude mixtures but their extensive delivery requires sophisticated procedures making terrorist mass attacks with BoNTs unlikely, though selected episodes cannot be excluded (Arnon et al. 2001; Bigalke and Rummel 2005; Cenciarelli et al. 2019). On the other hand, thanks to extensive scientific research that has unveiled the exceptional biological properties of BoNT, including exquisite neuroselectivity and specific enzymatic activity, together with limited diffusion from the site of injection (Carli et al. 2009; Eleopra et al. 2004), have been exploited for beneficial purposes, including human therapy and use as research tools for cell biology and physiology.

Investigations into the use of botulinum toxin type A1 for the treatment of hyperactive muscle disorders originated in the early ‘70 s (Scott et al. 1973; Scott 1980; Schantz and Johnson 1992). Building on these pioneer studies, BoNT/A1 was gradually expanded to the treatment of many movement disorders (Jankovic et al. 2017; Pirazzini et al. 2017 a) and, more recently, in esthetical medicine (Dover et al. 2018). It is produced by many companies and these products are formulated differently, have different manufacturing process, and demonstrate variable potencies and properties. For the description of the therapeutic use of BoNT/A1 we refer to the many reviews appeared in recent years (Jankovic 2017; Jost et al. 2015; Pirazzini et al. 2017; Choudhury et al. 2021). Here, we will focus on recent and emerging applications. Indeed, given its commercial success and clinical profile, significant efforts have been taken to extend the therapeutic use of BoNT through both indication discovery and novel product development (Maiarù et al. 2018).

## Novel therapeutic applications of botulinum neurotoxins

BoNTs have a much wider range of applications than originally understood (Dressler and Benecke 2003; Hallett et al. 2013; Jankovic 2017; Pirazzini et al. 2017). Indeed, in recent years, novel indications have emerged in many fields including dermatology, pain, osteoarthritis, wound healing and depression and this process is increasing.

### Dermatology

BoNTs can affect both sympathetic and parasympathetic functionality since acetylcholine is a neurotransmitter of the autonomic nervous system and the first non-motor indication was hyperhidrosis, initially approved in 2004. The strong efficacy and safety profile of BoNT-A1 for hyperhidrotic conditions has driven clinicians to evaluate its therapeutic potentiality in many other cutaneous diseases (Guida et al. 2018). Scar prevention, as well as vascular and inflammatory skin disorders, oily skin, alopecia, rhytides and cutaneous lesions, are some of the novel indications for BoNT in cosmetic and notably non-cosmetic aspects of dermatology (Martina et al. 2021; Naik 2021). These emerging clinical applications stem also from recent evidence that the BoNT/A1 receptors and intracellular targets are not unique for neurotransmission. The non-neuronal cells related to skin conditions that express BoNT/A-binding sites, and/or cleavage target SNAP25, include epidermal keratinocytes, mesenchymal stem cells from subcutaneous adipose, neutrophils and macrophages (Grando and Zachary 2018). Serotype BoNT/A1 can also elicit specific biological effects in dermal fibroblasts, sebocytes and vascular endothelial cells. Non-traditional applications of BoNT have been reported for the treatment of different dermatological conditions.

*Oil skin* is a common disorder, and available treatment options often provide unsatisfactory results. In a prospective study, Rose and Goldberg (2013) evaluated efficacy and safety of intradermal botulinum toxin for the treatment of oily skin in the forehead region in 25 subjects and reported a significantly lower sebum production with patient satisfaction. This efficacy seems to lie in the expression in sebaceous glands of muscarinic acetylcholine receptors that control sebocyte differentiation and sebum production and are possible targets of the neuromodulatory effects of BoNT. Moreover, Li et al. (2013) showed that human skin sebaceous glands in vivo and sebocytes in vitro express nicotinic acetylcholine receptor α7, and that acetylcholine increased lipid synthesis in a dose-dependent manner. More recent retrospective reviews have suggested the role of the intradermal injection of BoNT-A1 in decreasing sebum production and pore size with high patient satisfaction without significant side effects (Shuo et al. 2019). Intradermal BoNT-A1 injection may represent an innovative promising treatment for oily skin and other relevant dermatological problems, such as enlarged pores, acne, and seborrheic dermatitis (Martina et al. 2021).

*Rosacea* is a common inflammatory dermatosis characterized mainly by facial flushing and erythema. The onset of rosacea typically occurs after 30 years of age. It is estimated that approximately 2–5% of adults worldwide are affected. The effects of BoNT/A1 were studied in 15 rosacea patients. 15–45 IU of neurotoxin were injected into the face, resulting in a statistically significant erythema improvement and no adverse effects were mentioned (Bloom et al. 2015). BoNTs’ role in rosacea is not well understood and controlled, randomized studies are needed. However, a possible mechanism is that the erythema may fade away because local skin inflammation is reduced and controlled by BoNT robust suppression of inflammatory mediators such as calcitonin gene-related peptide (CGRP) and substance P (SP) (Naik et al. 2021). Moreover, given recent evidence suggesting a role for mast cells in the pathogenesis of rosacea via the activation and secretion of various immune mediators (Choi et al. 2019), a direct inhibitory effect of BoNT/A1 on mast cells degranulation after BoNT intradermal injection was proposed. Moreover, a topical preparation of BoNT/A1 was suggested for future studies (Choi et al. 2019).

#### Scar prevention and keloids

BoNT has been shown to be useful in scar prevention (Gassner et al. 2006; Ziade et al. 2013; Kim et al. 2014). Tension due to motion in postoperative scar is a crucial factor of scar hypertrophy and it can be prevented by BoNTs’ tension-relieving effect, as well as via its direct inhibitory effects on fibroblasts and TGFβ expression, the main regulator of hypertrophic scar formation. Moreover, BoNTs’ anti-inflammatory effect decrease the inflammatory phase of the wound healing process, which may help prevent scarring.

Keloids are aberrant tissue scarring occurring after the injury. Although intralesional corticosteroids remain the treatment of choice, recently intralesional BoNT-A1 have become an excellent alternative by reducing the levels of TGF-β1 and CTGF, which will ultimately attenuate fibroblast differentiation. Multiple studies have reported therapeutic success of BoNT-A1 in keloid cases with very high patient satisfaction with the use intralesional BoNT-A1 injections (Sohrabi and Goutos 2020).

#### Alopecia

Alopecia areata, androgenetic alopecia, cephalalgic alopecia and radiation-induced alopecia have all been treated with BoNT-A1 (Cutrer et al. 2010; Zhou et al. 2020; Melo et al. 2021). Blood flow is a primary determinant in follicular health and although the exact mechanism by which BoNT aids hair regrowth is uncertain, it has been proposed that BoNT-A1 injected into the scalp would decrease microvascular pressure by relaxing muscles thus improving oxygen supply to hair follicles (Melo et al. 2021). Although most studies show a clinical improvement in density or growth of hair and high patient satisfaction, further randomized clinical trials are necessary to determine the real effect of BoNT on hair growth.

#### Psoriasis

No placebo-controlled studies are available at the moment, thus impairing a clear evaluation of the efficacy of BoNT in this disease. However clinical improvement in inverse psoriasis following BoNT-A1 injections was documented (Zanchi et al. 2008; Aschenbeck et al. 2018). In a preclinical study in a mouse model of psoriasiform dermatitis, intradermal injections of BoNT-A1 lead to significant improvement over placebo and a reduction in skin lymphocyte infiltration (Ward et al. 2012). Moreover, the high concentration of nerve fibres in psoriatic skin present together with an increased level of CGRP and SP of sensory nerve origin lead to the hypothesis that the toxin inhibits CGRP- and SP-derived nerve release with clinical remission of the disease (Martina et al. 2021; Amalia et al. 2021).

### Pain

Increasing clinical evidence indicate that BoNT can offer an effective, long-lasting pain relief, and very few side-effects in a wide range of medical pain conditions (Go et al. 2021). Despite the rapid growth of the field, prophylactic treatment of chronic migraine is the only pain indication currently approved with the highest level of efficacy supported by several clinical trials and meta-analysis (Jackson et al. 2012; Talbet and Elnahry, 2022). Moreover, BoNT has been used off-label in pain related disorders such as osteoarthritis, neuropathic pain and lower back pain.

#### Osteoarthritis

Osteoarthritis (OA) is the most common form of arthritis in humans and OA-related joint pain is a major health concern. There is a great need to identify novel strategies to reduce the impact of this highly prevalent and debilitating condition and the clinical focus is on pain management and minimizing the functional impairment of the joint. Since the release of sensory neuropeptides such as SP, CGRP and neurokinin A appears to contribute to the pain sensation in OA (Khenioui et al. 2016), intra-articular BoNT administration may be able to directly reduce peripheral sensitization by inhibiting the release of these peptides, and indirectly reduce central sensitization. A recent comparative clinical study of the therapeutic effects of intra-articular botulinum neurotoxin versus physical therapy in knee osteoarthritis concluded that the use of BoNT/A1 can reduce pain and improve the function and quality of life in patients (Rezasoltani et al. 2021). However, more research is necessary to understand the mode of action and the behaviour of the BoNT when injected intra-articular (Hsieh et al. 2016). The established ability to produce neurotoxins by recombinant protein expression (Masuyer et al. 2014) and to modify them to create engineered neurotoxins with enhanced specificity for nociceptive nerve terminals could be developed in future for treating OA and other pain conditions.

#### Neuropathic pain

This form of human pain derives from damage or dysfunction of the peripheral or central nervous systems and failure of response to current analgesic medications is not uncommon. The data on BoNT/A1 are encouraging and indicate efficacy or probable efficacy in three major and common forms of neuropathic pain, namely, postherpetic neuralgia, posttraumatic neuralgia, and painful diabetic neuropathy. A systematic review of the therapeutic value of BoNT/A1 subcutaneous or intradermal injection for the therapy of trigeminal neuralgia concluded that this treatment is to be considered as an alternative option to the surgery (Morra et al. 2016). In a recent study the repeated (≥ 6 times) intra/subcutaneous injections of BoNT/A1 over 2 years for the treatment of severe post-herpetic neuralgia showed marked pain reduction and no adverse events. Adjunctive local BoNT/A1 injection seems to be a safe and effective therapy in long-term management for chronic neuropathic pain (Ri et al. 2021). The most recent systematic review and meta-analysis of randomized controlled studies added evidence that BoNT-A1 is safe for treating neuropathic pain, without notable side effects, and it is durable over an extended period (Datta Gupta et al. 2022).

### Depression

Depression is the most common mental disorder affecting millions of people worldwide. It is commonly treated with antidepressants and psychotherapies which have limited efficacy and many side effects (Helton and Lohoff 2015). Thus, alternative approaches for preventing and treating depression are urgently required. It was soon reported that a single injection of BoNT/A1 in the glabellar frown lines was able to improve symptoms in ten patients suffering of depressive disturbs (Finzi and Wasserman 2006). Recent meta-analysis and literatures indicate that clinical application of BoNT/A1 may have antidepressant properties (Li et al. 2021; Brin et al. 2020; Qian et al. 2020). Despite its safety and efficacy, the underlying therapeutic mechanisms of BoNT/A1 for depression is still elusive. There are some review article or meta-analysis that discussed efficacy and possible mechanisms (Qian et al. 2020; Schulze et al. 2021). The facial feedback hypothesis claims that mood improvement in BoNT/A1-treated patients comes from the neuroparalytic effect of the toxin that improves the proprioceptive perception, thus neutralizing the sadness by decreased frowning (Magid et al. 2014, 2015). Social feedback hypothesis indicated that happy facial expressions obtains positive social feedback and improves mood. An additional hypothesis posits that facial injection of BoNT/A1 causes structure or function changes in the brain to alleviate depression, possibly by upregulating brain derived neurotrophic factor (BDNF) expression in the brain (França and Lotti, 2017). Indeed, reduction of depressive symptoms was reported to continue after BoNT/A1 cosmetic effect was ceased, tempting to speculate a possible direct effect of the toxin on central circuits. Although both clinical and preclinical studies have indicated that BoNT/A1 therapy may be an effective treatment option for depression, further investigations are needed to improve our understanding of the therapeutic mechanisms of BoNT/A1 for this condition.

## Novel potential therapeutics based on botulinum neurotoxins

Recently, a BoNT/E1, produced with recombinant methods in *E. coli*, was assayed in the human *Extensor Digitoris Brevis* muscle, which is a test muscle for BoNT, and it was found to show paralysis after 1–2 days, depending on the dose and this lasted for 2–7 weeks again depending on the dose (Pons et al. 2019). These preliminary experiments indicate that this novel therapeutics may find application in those conditions requiring a short duration of paralysis such as initial test of BoNT efficacy when critical situations need to be addressed, scar prevention, better recovery after displaced bone fractures, etc.

Some human pathological conditions are caused by hypersecretion of active biological molecules contained in vesicles whose plasma membrane fusion is mediated by SNAP-23 rather than SNAP-25. These exocytosis events include the release of chemokines or vasoactive amines in mast cells and other inflammatory mediators from neutrophils (Martin-Martin et al. 2000; Frank et al 2011). To treat these diseases, the metalloprotease domain of BoNT/E1 was mutated to become capable of cleaving SNAP-23 in addition to SNAP-25. Such modified metalloprotease domain was introduced in HeLa cells and shown to inhibit the release of IL-8 and mucin providing a first proof of principle that this line of research can be further pursued (Chen and Barbieri 2009). Additional mutagenesis studies performed on BoNT/A1 L domain led to the production of a four-residues BoNT/A mutant that efficiently cleaves SNAP-23 in neurons (Sikorra et al. 2021). The authors proposed further modifications on the HC-C binding part to render it specific for immune cells and a candidate therapeutic for immune diseases caused by hypersecretion of immune messenger molecules (Sikorra et al. 2021).

Perhaps the most innovative study in the search of novel therapeutics based on botulinum neurotoxins is a basic science study of molecular evolution in vitro which described an original phage-assisted method of continuous production of variants of the metalloprotease L domain of BoNTs (Blum et al. 2021). These variants were submitted to negative and positive selection performed at the same time generating a very powerful method to identify novel metalloproteases with a novel target selectivity. The authors first focused on the L chain of BoNT/X which cleaves different isoforms of VAMPs and identified a mutant which cleaves only VAMP-4 whilst retaining the capability of forming a BoNT molecule with the native H chain. They then evolved the L domain of BoNT/F1 to cleave selectively the VAMP-7 isoform leaving untouched VAMP-1 thus reversing the selectivity of wild-type BoNT/F1. Even more remarkable was the generation of a BoNT/E metalloprotease L domain targeting the suppressor oncogene PTEN (phosphatase and tensin homolog) whilst leaving intact its original target SNAP-25. This study is a technological breakthrough that opens an avenue to treating the many neurodegenerative diseases due to alterations of specific intraneuronal proteins.
